# Accuracy of endoscopic ultrasonography for diagnosing ulcerative early gastric cancers

**DOI:** 10.1097/MD.0000000000003955

**Published:** 2016-07-29

**Authors:** Jin-Seok Park, Hyungkil Kim, Byongwook Bang, Kyesook Kwon, Youngwoon Shin

**Affiliations:** Digestive Disease Center, Department of Internal Medicine, Inha University School of Medicine, Incheon, South Korea.

**Keywords:** early gastric cancer, endoscopic ultrasonography, endoscopy, staging, ulcer

## Abstract

Although endoscopic ultrasonography (EUS) is the first-choice imaging modality for predicting the invasion depth of early gastric cancer (EGC), the prediction accuracy of EUS is significantly decreased when EGC is combined with ulceration.

The aim of present study was to compare the accuracy of EUS and conventional endoscopy (CE) for determining the depth of EGC. In addition, the various clinic-pathologic factors affecting the diagnostic accuracy of EUS, with a particular focus on endoscopic ulcer shapes, were evaluated.

We retrospectively reviewed data from 236 consecutive patients with ulcerative EGC. All patients underwent EUS for estimating tumor invasion depth, followed by either curative surgery or endoscopic treatment. The diagnostic accuracy of EUS and CE was evaluated by comparing the final histologic result of resected specimen. The correlation between accuracy of EUS and characteristics of EGC (tumor size, histology, location in stomach, tumor invasion depth, and endoscopic ulcer shapes) was analyzed. Endoscopic ulcer shapes were classified into 3 groups: definite ulcer, superficial ulcer, and ill-defined ulcer.

The overall accuracy of EUS and CE for predicting the invasion depth in ulcerative EGC was 68.6% and 55.5%, respectively. Of the 236 patients, 36 patients were classified as definite ulcers, 98 were superficial ulcers, and 102 were ill-defined ulcers, In univariate analysis, EUS accuracy was associated with invasion depth (*P* = 0.023), tumor size (*P* = 0.034), and endoscopic ulcer shapes (*P* = 0.001). In multivariate analysis, there is a significant association between superficial ulcer in CE and EUS accuracy (odds ratio: 2.977; 95% confidence interval: 1.255–7.064; *P* = 0.013).

The accuracy of EUS for determining tumor invasion depth in ulcerative EGC was superior to that of CE. In addition, ulcer shape was an important factor that affected EUS accuracy.

## Abstract 2

Supplemental Digital Content is available in the text

## 1 Introduction

Early gastric cancer (EGC) is defined as gastric cancer confined to the mucosal or submucosal layers, regardless of the presence of lymph node metastasis.^[1]^ Endoscopic treatment, especially endoscopic submucosal dissection (ESD), is currently the widely accepted standard treatment for EGC in Korea and Japan, as it is minimally invasive and effective in curative management.^[2,3]^ In addition, its excellent feasibility and long-term efficacy have been proven in a large-scale study.^[4]^ On the basis of surgical data, the indications for endoscopic resection of EGC have been extended to treat minute submucosal cancer and ulcerative EGC.^[5]^

Endoscopic ultrasonography (EUS) is the most reliable nonsurgical method available for pretreatment staging of EGC, with an accuracy rate from 80% to 90%.^[6]^ With EUS, patient selection for curative endoscopic treatment can be made using the classification of EGC as mucosal or submucosal cancer, based on detecting the extent of the ultrasonographic changes.

However, previous studies reported that EUS accuracy is significantly decreased (to 55–70%) when EGC is combined with ulceration.^[7–9]^ The main reason for this inaccuracy is generally considered to be fibrosis of the ulcer, which is seen on EUS as a hypoechoic lesion, similar to tumor invasion; EUS can thus not clearly distinguish it. This inaccuracy raises the question of the necessity of EUS in EGC with ulceration; some authors have reported that EUS is not necessary to determine the endoscopic resection for EGC, and that conventional endoscopy may be sufficient for determining the optimal therapeutic strategy.^[7,8]^

Unfortunately, the feasibility of EUS for ulcerative EGC is yet to be properly evaluated. There have been a few studies on EUS accuracy for ulcerative EGC; however, these were not large-scale studies, and EGC with ulceration represented only a small part of the total EGC cases. In addition, these previous reports mainly focused on the relationships between EUS accuracy in predicting the T stage of EGC and the degree of endoscopic ulcer depth; there are no studies focusing on the relationship between EUS accuracy and ulcer shape. As part of gastric cancer's natural history, most EGC with ulceration cases are believed to go through a “malignant cycle,” consisting of ulceration followed by healing and reulceration.^[10]^ Therefore, ulceration in EGC would present a variety of endoscopic ulceration shapes, and the accuracy of EUS for predicting cancer invasion can be affected by these shapes during EUS examination. We therefore conducted a study to compare EUS and conventional endoscopy for T-staging in patients with ulcerative EGC. We also attempted to determine whether the endoscopic ulcer shapes of EGC are potential risk factors that affect the accuracy of EUS in determining tumor invasion depth. Finally, we analyzed various clinicopathologic factors to evaluate whether they affected the diagnostic accuracy of EUS in EGC with ulceration.

## 2 Methods

### 2.1 Study design and study population

We retrospectively reviewed the medical records of consecutive patients with ulcerative EGC. The inclusion criteria were age >18 years; EGC confirmed using conventional endoscopy and biopsies; received EUS for pretreatment staging; and received curative treatment by either endoscopic resection or by standard surgical intervention. Exclusion criteria were poor-quality endoscopic image for lesion characterization; ulcer scars without a definite endoscopic ulcers; not a definite endoscopic ulcer; and inadequate medical record.

We enrolled 236 patients with ulcerative EGC who underwent EUS between January 2005 and December 2014. Endoscopic staging was divided into mucosal and submucosal invasion on the basis of marked ulcer depression, marginal elevation, or an interrupted/enlarged fold. The EUS staging was categorized into mucosal, submucosal, and advanced depending on the invasion depth seen on EUS. The patients’ medical records were analyzed and the following information extracted: clinical characteristics, conventional endoscopic information, histologic information of EGC, and EUS findings. This retrospective study was performed at the Inha University Hospital, Incheon, South Korea. The study was approved by the hospital's institutional review board.

### 2.2 Endoscopic findings

**Figure 1:**
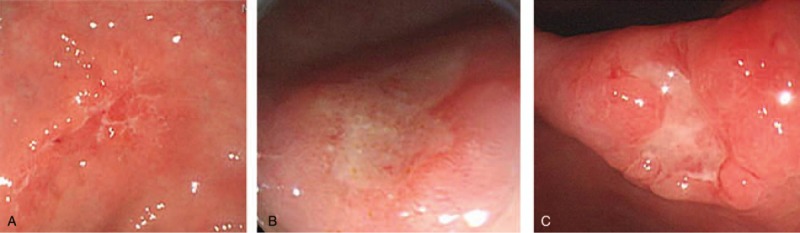
Endoscopic images of ulcerative early gastric cancers. (A) Ill-defined ulcer, showing a slightly depressed lesion with an irregular ulcer shape, or an ulcer size <1 cm. (B) Superficial ulcer, showing a slightly depressed lesion with a remarkable ulcer margin and an ulcer size ≥1 cm. (C) Definite ulcer, with a concave ulcer base and an ulcer depth that was greater than the thickness of adjacent mucosal surface.

We reviewed the endoscopic factors, including ulcer shape and depth, and lesion size and location. The conventional endoscopes used in this study were either GIF-H260 or GIF-Q260 (Olympus, Tokyo, Japan). Ulcerative EGC was defined as an EGC combined with an endoscopic ulcer (IIc and III); we excluded ulcer scars (IIb) without exudative bases.^[11]^ The endoscopic ulcer was classified when endoscopic findings met the following criteria: ill-defined ulcer, with slightly depressed lesion and exudative base showing either an irregular ulcer shape or size <1 cm; superficial ulcer, with a slightly depressed lesion and an exudate base showing a remarkable ulcer margin and a size ≥1 cm; and definite ulcer, with a concave ulcer base, and ulcer depth greater than the thickness of the adjacent mucosal surface (Fig. 1). To denote the location of the lesion, the stomach was divided into 3 equal portions: upper, middle, and lower. The endoscopic criteria used for T staging were as follows: mucosal cancer (a shallow ulcer with a depressed lesion) or submucosal cancer (an irregular-based ulcer with marginal mucosal elevation, a marked depressed ulcer, or an ulcer with interrupted enlarged folds).^[12]^

### 2.3 EUS findings

**Figure 2:**
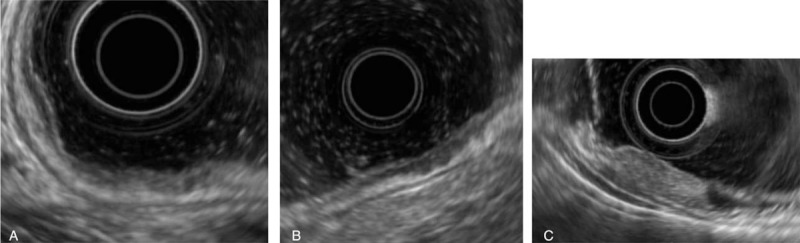
Endoscopic ultrasound images of ulcerative early gastric cancers. (A) Endoscopic ultrasonography (EUS)-mucosa, a hypoechoic tumor disrupted the muscularis mucosa, but with the hyperechoic layer 3 (submucosa) intact. (B) EUS-submucosa, a hypoechoic tumor invading the submucosa. (C) EUS-advanced lesion, a hypoechoic tumor invading the muscularis propria.

All EUS staging was performed using a radial array endoscope (GF-UE260-AL5; Olympus Medical Systems Co Ltd, Tokyo, Japan) or a miniature ultrasound probe (miniprobe, Olympus UM-2R, 12 MHz; Olympus Medical System Co Ltd) by 3 experienced endosonographers, who had previously performed more than 2000 examinations. The conventional endoscopy and EUS were performed by same endoscopy. On the EUS images, the depth of cancer invasion was determined as the deepest of the sonograophic 5 layers in the gastric wall that was disrupted from tumor. The EUS criteria used for T staging was as follows: mucosal (EUS-M): the neoplasm had infiltrated the first and/or second layer but not the third layer; submucosal (EUS-SM): either the neoplasm infiltrated the third layer but not the fourth layer or it showed hypoechoic focal thickening of the third layer; and advanced lesion (EUS-A): the neoplasm infiltrated the fourth layer or deeper (Fig. 2).^[7]^

### 2.4 Histopathologic staging

Endoscopic resection or gastrectomy was performed within 2 weeks of performance of EUS. Histopathologic examination of the resected specimens was carried out in parallel 5 mm thick sections stained with hemotoxylin and eosin. Histological findings, such as tumor size, depth of invasion, degree of differentiation, lymphatic and vascular involvement, perineural invasion, and lymph node metastasis, were reviewed. Submucosally invasive cancer in surgically resected specimens is classified as SM1 (upper third of the submucosal layer), SM2 (middle third), or SM3 (lower third) according to the degree of invasion into the submucosa. In the endoscopically resected cases, invasion depth was also classified into 2 groups: SM1 (penetration into the submucosal layer <500 μm from the muscularis mucosa), and SM2 (penetration >500 μm).^[9,13]^ When the tumor invaded beyond submucosal layer, the lesions were classified as “advanced.”

### 2.5 Statistical analysis

The histopathology of the resected specimens was the gold standard to which we compared the results of the EUS and conventional endoscopy T staging. The continuous variables were presented as the mean and standard deviation (SD). We calculated the sensitivity, specificity, positive predictive value (PPV) and negative predictive value (NPV), and the accuracy of the conventional endoscopy and EUS assessment for submucosal cancer. The EUS accuracy in relation to the clinicopathological factors was assessed using either the *x*^2^ test or Fisher exact test. For the preliminary evaluation, univariate logistic regression analysis was performed to determine whether particular factors influenced the accuracy of EUS staging. A multivariate logistic regression analysis was performed to test the outcomes of the univariate logistic regression analysis. The odds ratios (ORs) and the 95% confidence intervals (CIs) were presented. A *P* value of <0.05 was considered statistically significant. The statistical calculations were performed using SPSS version 19.0 for Windows software (SPSS Inc, Chicago, IL).

## 3 Results

### 3.1 Patient demographics

The patients’ baseline characteristics and the histologic results are summarized in Table 1. The mean patient age was 61.81 (10.45) years; the male:female ratio was about 2:1 (165:71 cases). A total of 111 patients with ulcerative EGC were curatively treated using ESD, and 125 patients underwent surgical resection as the initial treatment modality. In addition, 21 patients received surgical resection after ESD for the following reasons: SM2/SM3 invasion, lymphatic invasion, and lateral resection margin positivity. The histologic results confirmed mucosal cancer in 134 patients (56.8%), SM1 in 33 (14%), SM2 or SM3 in 54 (22.8%), and proper muscle layer involvement in 15 (6.4%). The mean tumor size was 2.74 cm (SD: 1.7; 95% CI: 2.53–2.96). Histologically, the most common type of tumor was the moderate differentiated type (28%).

**Table 1:**
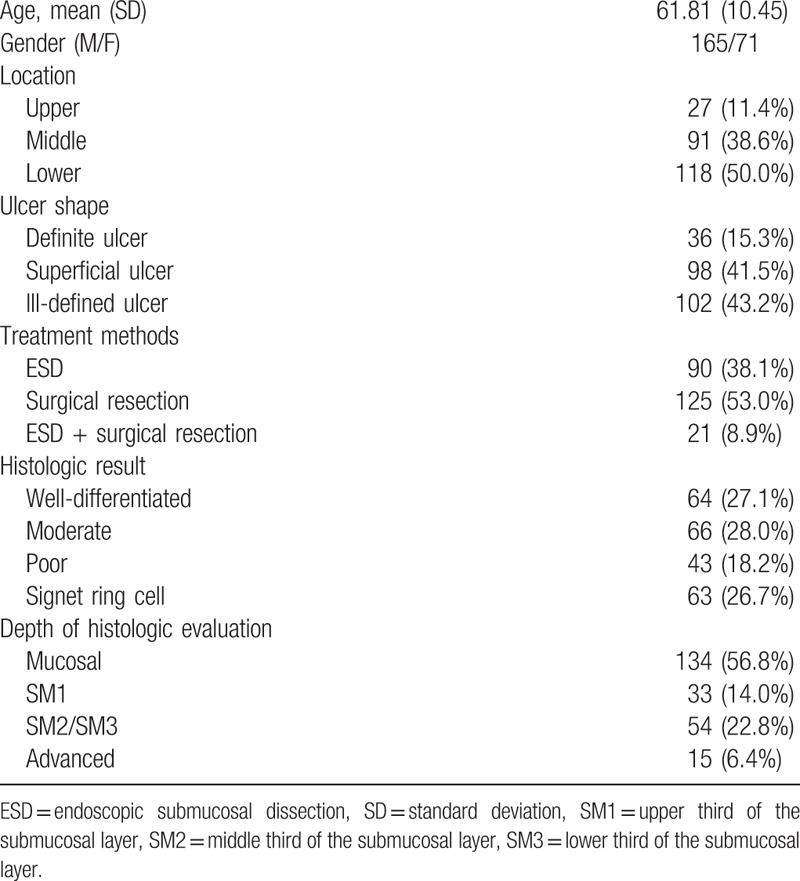
Baseline characteristics of patients and early gastric cancer.

### 3.2 Comparison of T-staging accuracy for both EUS and conventional endoscopy in ulcerative EGC

**Figure 3:**
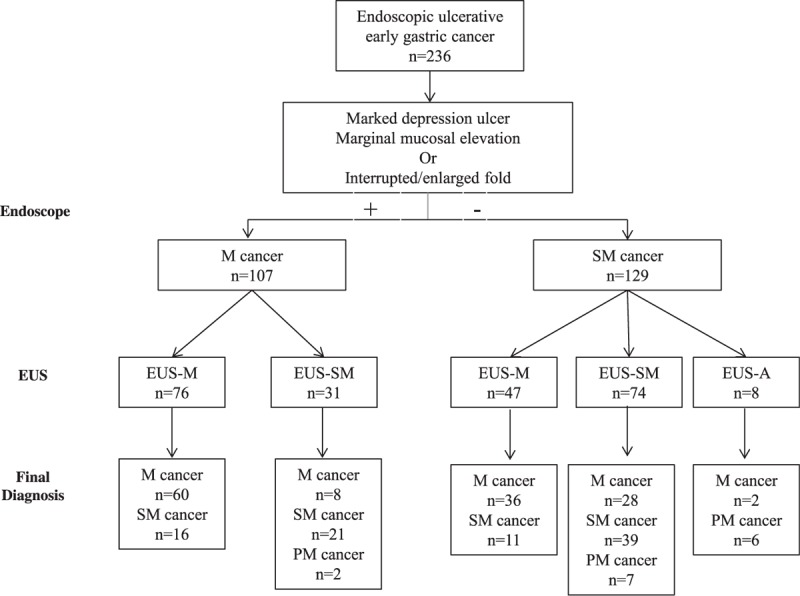
Distribution of patients on the basis of receipt of conventional endoscopy or endoscopic ultrasonography, and the histologic results.

The overall accuracy of EUS staging was 68.6% (162 of 236; 95% CI: 0.63–0.75) and that of conventional endoscopy was 55.5% (131 of 236; 95% CI: 0.49–0.61) (*P* < 0.001). According to conventional endoscopic imaging, 107 and 129 patients with ulcerative EGC were classified with mucosal cancer and submucosal cancer, respectively. Within the mucosal cancer group, 31 patients were diagnosed as EUS-SM following EUS staging. Out of these 31 patients, 23 had the histological diagnosis of either submucosal (n = 21) or proper muscle (n = 2) invasion, while the remaining patients were diagnosed with mucosal cancer (n = 8). Of the 129 submucosal cancers seen using conventional endoscopic staging, 47 patients were reclassified with EUS-M through EUS staging, and 31 received the histological diagnosis of mucosal cancer after curative resection (Fig. 3). The diagnostic accuracy, sensitivity, and specificity of EUS for submucosal cancer were 72.5%, 73.5%, and 71.6 respectively, and the PPV and NPV was 66.4% and 78%, respectively. These results were more accurate than those seen using conventional endoscopy, in which accuracy, sensitivity, specificity, and PPV and NPV were 55.5%, 61.7%, 50.7%, 48.8%, and 63.5%, respectively. Among the 105 EUS-SM cancers, 45 were incorrectly staged: 36 (34.3%) were overstaged and 9 (8.7%) were understaged. When at least 1 of 2 endoscopic images postulated submucosal infiltration, the sensitivity of submucosal invasion diagnosis, including submucosal cancer and proper muscle invasion, improved to 84.3% (86 of 102); however, specificity decreased to 52.2% (70 of 134).

### 3.3 Diagnostic accuracy of EUS based on ulcer shapes

The ulcerative EGC, classified according to the shape of ulcers, showed 102 cases (43.2%) with ill-defined ulcers, 98 (41.5%) with superficial ulcers, and 36 (15.3%) with definite ulcers. The histologic results, according to the shapes of endoscopic ulcers, were as follows: for ill-defined ulcers, 57 mucosal cancers (55.9%), 35 submucosal cancers (34.3%), and 10 advanced cancers (9.8%); for superficial ulcers, 60 mucosal cancers (61.2%), 36 submucosal cancers (36.7%), and 2 advanced cancers (2%); and for definite ulcers, 17 mucosal cancers (47.2%), 16 submucosal cancers (44.4%), and 3 advanced cancers (8.3%) (Table 2). The endoscopic ulcer shape was significantly related to the T-staging accuracy of EUS (*P* < 0.01). Eighty (81.6%; OR: 3.035; 95% CI: 1.64–5.61; *P* < 0.01) of 98 EGC with superficial ulcers could be correctly classified using EUS staging. On the contrary, the diagnostic accuracies of EUS for definite ulcers (55.6%; OR: 0.51; 95% CI: 0.25–1.05; *P* = 0.07) and for ill-defined ulcer (60.8%; OR: 0.53; 95% CI: 0.30–0.92; *P* = 0.02) were less accurate than that for superficial ulcers (Table 3). In the logistic analysis, EUS staging was more accurate for the superficial ulcer than for the other ulcer types (OR: 2.98; 95% CI: 1.26–7.06; *P* = 0.01). In multivariate analysis, superficial ulcer was also significantly associated with the T-staging accuracy of EUS (OR: 3.56; 95% CI: 1.55–8.18; *P* < 0.01) (Table 4).

**Table 2:**
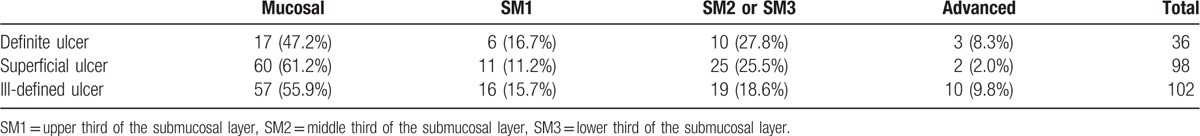
Histologic invasion depth based on ulcer shape.

**Table 3:**
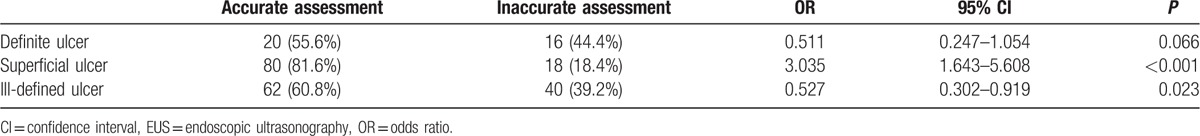
Analysis for correct diagnosis of EUS depending on ulcer shape.

**Table 4:**
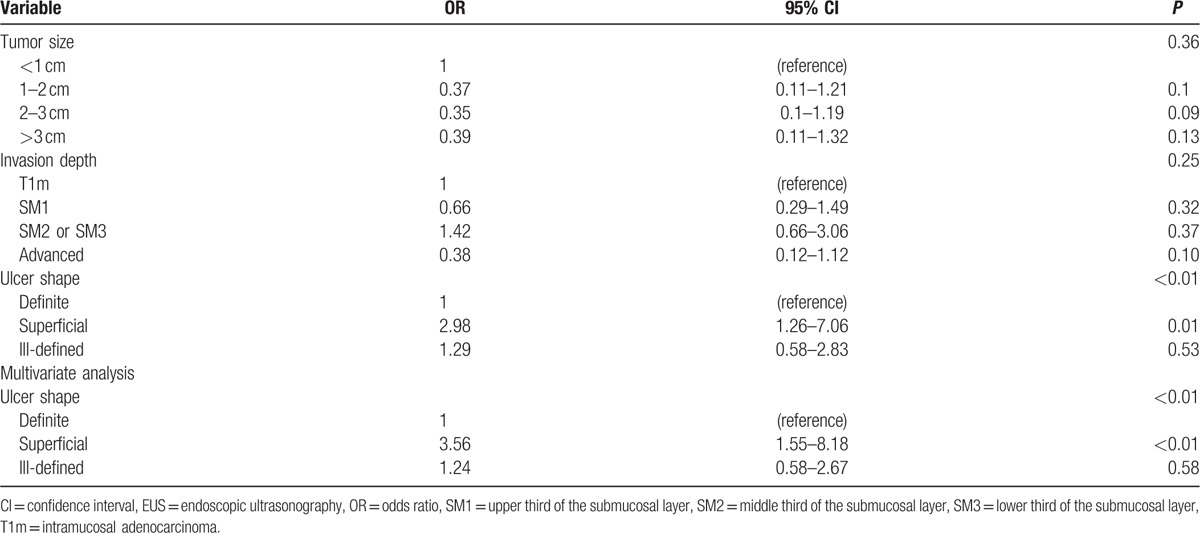
Univariate and multivariate analysis for the clinical factors affecting EUS accuracy.

### 3.4 Clinicopathologic factors affecting EUS accuracy

The EUS accuracy tended to decline according to the depth of the tumor invasion. The accuracy for mucosal cancer was 71.6%, but for proper muscle invasion was only 40% (*P* = 0.023). An increase in tumor size also tended to decrease EUS accuracy; increased tumor size was inversely related to EUS accuracy. The accuracy was 87.9% for tumors of <1 cm and 63.5% for tumors of >3 cm (*P* = 0.03) (Table 5). However, logistic analysis showed that the tumor invasion depth (*P* = 0.25) and tumor size (*P* = 0.36) were not associated with EUS accuracy. Other clinicopathologic factors, such as gender (*P* = 0.49), age (*P* = 0.15), location in stomach (*P* = 0.28), and histological type (*P* = 0.1), were not statistically significant. In the multivariate analysis, only superficial ulcer (OR: 3.56; 95% CI: 1.55–8.18; *P* < 0.01) remained as an independent factor affecting EUS accuracy.

**Table 5:**
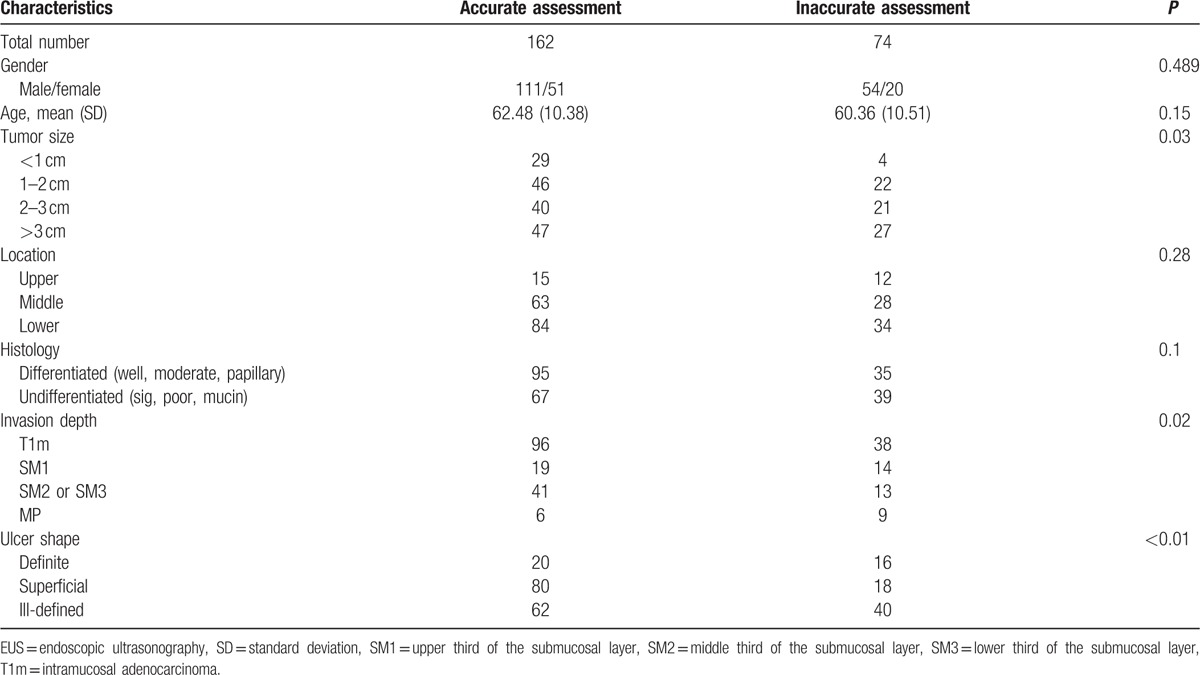
The clinical factors affecting EUS accuracy in assessing tumor invasion depth.

## 4 Discussion

The present study indicates that EUS shows superior accuracy for T staging of ulcerative EGC when compared with conventional endoscopy. The overall diagnostic accuracy of EUS and conventional endoscopy in ulcerative EGC was 68.6% and 55.1%, respectively, with significant differences between the 2 modalities (*P* < 0.01). EUS is generally considered the most accurate method for T staging of EGC because it can delineate the individual gastric wall layers with histologic correlates.^[6]^ However, some authors have refuted the use of EUS for T staging in EGC, as the diagnostic accuracy of EUS has been variable and inconsistent in terms of distinguishing mucosal lesions from submucosal invasion, especially in ulcerative EGC.^[14,15]^ Akashi et al^[14]^ studied 267 patients with gastric adenocarcinoma, including 29 with ulcerous changes, to evaluate the effect of ulcerous change on EUS diagnostic accuracy for the invasive depth of EGC. In their results, ulcerous change (OR: 9.903; 95% CI: 3.842–28.167; *P* < 0.0001) was a factor that caused incorrect diagnosis when using EUS, and they concluded that the EUS accuracy for tumor staging was not sufficient for lesions with ulcerous changes. Another prospective study also reported similar results.^[7]^ Their study examined 388 patients with EGC, including 30 patients of ulcerative EGC, and found that the presence of ulceration was the factor that could cause unsatisfactory EUS accuracy (63.3%), and that EUS did not show a PPV for T staging over the use of conventional endoscopy alone.

However, these studies were somewhat limited by the various types of EGC, including a relatively small proportion of subjects with ulcerative EGC. Our study has the advantage in this respect; we only enrolled cases of EGC accompanied by endoscopic ulcer, had a large sample size, and provided valuable data in predicting EUS accuracy for ulcerative EGC. In our study, the overall accuracy of EUS for ulcerative EGC was 68.6%, which is similar to previous reports.^[9,16]^ However, the EUS accuracy rate for distinguishing between mucosal cancer and submucosal invasion improved to 77.1%. Although this accuracy rate was not a satisfactory result in our estimation, this result is still important, particularly when compared with those seen using conventional endoscopy (55.5%). The diagnostic accuracy of conventional endoscopy for ulcerative EGC has not yet been fully established, but limited data have established the accuracy of conventional endoscopy for ulcerative EGC at about 65%.^[8,13]^ Compared with these results, the diagnostic accuracy for conventional endoscopy in our study was low. The reason for this inaccuracy is unclear; we assumed it was related to the ambiguous criteria used to distinguish mucosal cancer from submucosal invasion when using conventional endoscopy.

The endoscopic criteria used in our study, such as marginal mucosal elevation, marked depressed ulcers, or ulcers with interrupted enlarged folds, were very subjective. Although all our endoscopic evaluations were performed by 3 experienced endoscopists, the results of classification differed somewhat from one another, and thus the results were not very accurate. Approximately 50% of cases with 1 of these 3 findings were finally diagnosed with mucosal cancer in histologic examination (Supplementary Table). In particular, the 47.2% of patients with definite ulcer, which is generally considered as the endoscopic feature of submucosal invasion, actually showed mucosal cancer in histologic examination. On the contrary, EUS staging based on changes in the sonographic layer were more objective, as the usual 5-layer normal architecture of the gastric wall can be easily distinguished in EUS, and the interpretation of tumor invasion also can be found without differences in each endosonographer's opinions. Thus, the present study suggested that the diagnostic accuracy of the depth of tumor invasion can be improved using EUS.

The remarkable outcome of our study was demonstrating that EUS accuracy for ulcerative EGC is significantly different on the basis of the endoscopic ulcer shape. To date, the specific endoscopic ulcer shapes of EGC that can predict EUS accuracy have not been defined. The only study on this subject found that endoscopic ulcers decrease EUS accuracy in predicting invasion depth.^[17]^ However, endoscopic ulcers have a variety of shapes, and the ulcer fibrosis or inflammation is also different according to the nature of ulcer. Therefore, we focused on the ulcer shape on EGC to determine whether the endoscopic features of ulcers can affect the accuracy of EUS staging. We defined 3 categories of ulcerative EGC: definite ulcer, superficial ulcer, and ill-defined ulcer, with the ulcer shapes classified on the basis of depth and shape.^[11]^ Definite ulcer was defined as such when the ulcer base was concave, and the ulcer depth was greater than the thickness of adjacent mucosal surface, which is same indication as the type III ulcer using the Paris endoscopic classification of superficial neoplastic lesions. Both superficial ulcers and ill-defined ulcers were type IIc ulcers, and they were distinguished on the basis of the ulcer shape. In our results, the ulcer shapes (*P* < 0.01) rather than with ulcer depths (*P* = 0.36) were more associated with EUS accuracy. Superficial ulcers were especially independently correlated with EUS accuracy (81.6%), which seemed to be enough of a satisfying result to consider EUS staging as a reliable method for predicting the EGC depth. It is likely that the superficial ulcer in EGC is related to the lesser presence of ulcer fibrosis or inflammation than in the other types of ulcerative EGC, because a large portion of EGCs with superficial ulcers were diagnosed as mucosal cancers (61.2%). However, the retrospective cohort design of our study made it difficult to evaluate this possibility. A larger-scale prospective study on this issue is warranted.

In the current study, the generally accepted factors that affect the EUS accuracy in EGC, such as tumor location in stomach, histology type, tumor size, and tumor invasion depth, were not related to EUS accuracy in ulcerative EGC. Although tumor size (*P* = 0.03) and tumor invasion depth (*P* = 0.02) was related with accuracy in the univariate analysis, they did not show correlation in multivariate analysis. The ulcer shape was the only factor that was related to EUS staging accuracy in multivariate analysis. Therefore, we recommend the use of EUS staging for accurate diagnosis of tumor invasion, even when EGC is accompanied by endoscopic ulcer; however, the clinician should be concerned by the fact that both definite and ill-defined ulcers carry a risk of misdiagnosis compared with superficial ulcers.

Although EUS staging has shown the remarkable results for accurate diagnosis of tumor invasion, EUS staging was deemed insufficient for differential diagnosis of mucosal cancer or submucosal invasion. Ulcer fibrosis or peritumoral inflammation sometimes makes it difficult to distinguish between mucosal cancer and submucosal cancer.^[8]^ The EUS findings for tumor invasion were similar with those for ulcer fibrosis, so fibrosis invasion is not adequately differentiated from true tumor invasion when using EUS. Therefore, the subjective assessments of endoscopists intervene in the interpretation of these lesions, which may lead to inaccuracies in EUS staging. In our study, 35 (34.3%) of 105 patients with EUS-SM were overstaged and 8.6% were understaged. We believe that this subjective assessment is related to this misreading. To overcome this problem, we introduced many strategies, such as contrast-enhanced EUS^[18]^ and pattern analysis.^[16]^ However, the differentiation between tumor invasion and ulcer fibrosis remains a challenging problem.^[9]^ Proper management of EGC should always begin with an accurate endoscopic evaluation.

We also found that conventional endoscopy reliably provided additional information in distinguishing mucosal cancer from submucosal invasion: the 8 mucosal cancers and the 11 submucosal cancers incorrectly identified as submucosal cancer and mucosal cancer, respectively, by EUS staging were subsequently correctly identified by conventional endoscopic classification. In addition, when submucosal invasion was assumed to be present if either conventional endoscopy or EUS postulated it, sensitivity increased to 84.3% (86 of 134). This suggests that the combined use of EUS and conventional endoscopy is an effective tool for differentiating submucosal cancer from mucosal cancer. However, these combined tools are not suitable for making treatment strategy decisions because of their low specificity (52.2%). Therefore, we proposed that conventional endoscopic staging should complement EUS staging; after close investigation with EUS, conventional endoscopy will provide clear information about the tumor invasion depth.

There were some limitations in the present study. First, this was a nonrandomized retrospective cohort study at a single center, raising the possibility of selection bias; therefore, our results may not be generalizable. Second, it was not possible to determine why superficial ulcers were associated with high accuracy rates in EUS staging. We assumed that the level of ulcer fibrosis would be related to this result, but this hypothesis should be tested using a prospective study.

In conclusion, when compared with conventional endoscopy, EUS was superior in predicting tumor invasion in ulcerative EGC. We recommend the use of EUS as a complement to conventional endoscopy in predicting the tumor invasion depth in ulcerative EGC. In addition, the ulcer shape on EGC was an important factor affecting the EUS accuracy.

## Supplementary Material

Supplemental Digital Content
