# Supplementary material for: Accuracy of endoscopic ultrasonography for diagnosing ulcerative early gastric cancers
Source: Medicine (Baltimore). 2016 Jul 29;95(30):e3955. doi: 10.1097/MD.0000000000003955 (PMC5265809; doi:10.1097/MD.0000000000003955)
Supplement: Supplemental Digital Content [file medi-95-e3955-s001.doc]

| Supplementary. The histologic invasion depth depending on the endoscopic image signs of submucosal (SM) invasion | | | | | | |
| --- | --- | --- | --- | --- | --- | --- |
| Endoscopic criteria for SM invasion | Mucosal | SM1 | SM2 | SM3 | Advanced | Total |
| Marked depressed ulcer | 17 (47.2%) | 6 (16.7%) | 4 (11.1%) | 6 (16.7%) | 3 (8.3%) | 36 |
| Marginal mucosal elevation | 55 (49.5%) | 12 (10.8%) | 14 (12.6%) | 18 (16.2%) | 12 (10.8%) | 111 |
| Interrupted enlarged folds | 28 (43.1%) | 9 (13.8%) | 7 (10.8%) | 12 (18.5%) | 9 (13.8%) | 65 |

SM1 indicates upper third of the submucosal layer; SM2, middle third of the submucosal layer; and SM3, lower third of the submucosal layer.
